# 

**Published:** 1918-12

**Authors:** 


### ANNOUNCEMENTS

---

*Atlanta—American Institute of Dental Teachers—January*—The next annual meeting of the American Institute of Dental Teachers will be held at Hotel Piedmont, Atlanta, Georgia, January 28, 29 and 30, 1919.

Papers on the teaching of war dentistry and an exhibit of war appliances will be the main features, and along with these will be the usual papers on teaching methods.

All persons interested are cordially invited.—ABRAM HOFFMAN, *Sec.*, 381 Linwood Ave., Buffalo, N. Y.

---

*Dental Protective Association*—The annual meeting of the Dental Protective Association will be held at the Palmer House in Chicago, on the third Monday of December, at four p. m. Report of the officers for the year and election of a new board of directors will be the order of business. All members are urged to attend.—J. G. REID, *President.*

